# ASaiM-MT: a validated and optimized ASaiM workflow for metatranscriptomics analysis within Galaxy framework

**DOI:** 10.12688/f1000research.28608.1

**Published:** 2021-02-11

**Authors:** Subina Mehta, Marie Crane, Emma Leith, Bérénice Batut, Saskia Hiltemann, Magnus Ø Arntzen, Benoit J. Kunath, Francesco Delogu, Ray Sajulga, Praveen Kumar, James E. Johnson, Timothy J. Griffin, Pratik D. Jagtap

**Affiliations:** 1 University of Minnesota, Twin Cities, MN, 55455, USA; 2 Department of Bioinformatics, University of Freiburg, Georges-Köhler-Allee 106, Freiburg, Germany; 3 Department of Pathology, Erasmus Medical Center, Rotterdam, The Netherlands; 4 Norwegian University of Life Sciences, Ås, 1430, Norway

**Keywords:** Galaxy, metatranscriptomics, microbiome, functional analysis

## Abstract

The Human Microbiome Project (HMP) aided in understanding the role of microbial communities and the influence of collective genetic material (the ‘microbiome’) in human health and disease. With the evolution of new sequencing technologies, researchers can now investigate the microbiome and map its influence on human health. Advances in bioinformatics methods for next-generation sequencing (NGS) data analysis have helped researchers to gain an in-depth knowledge about the taxonomic and genetic composition of microbial communities. Metagenomic-based methods have been the most commonly used approaches for microbiome analysis; however, it primarily extracts information about taxonomic composition and genetic potential of the microbiome under study, lacking quantification of the gene products (RNA and proteins). Conversely, metatranscriptomics, the study of a microbial community’s RNA expression, can reveal the dynamic gene expression of individual microbial populations and the community as a whole, ultimately providing information about the active pathways in the microbiome.  In order to address the analysis of NGS data, the ASaiM analysis framework was previously developed and made available via the Galaxy platform. Although developed for both metagenomics and metatranscriptomics, the original publication demonstrated the use of ASaiM only for metagenomics, while thorough testing for metatranscriptomics data was lacking.  In the current study, we have focused on validating and optimizing the tools within ASaiM for metatranscriptomics data. As a result, we deliver a robust workflow that will enable researchers to understand dynamic functional response of the microbiome in a wide variety of metatranscriptomics studies. This improved and optimized ASaiM-metatranscriptomics (ASaiM-MT) workflow is publicly available via the ASaiM framework, documented and supported with training material so that users can interrogate and characterize metatranscriptomic data, as part of larger meta-omic studies of microbiomes.

## Introduction

Understanding the role of microbiome in patho-physiological conditions such as inflammatory diseases, obesity, and cancer has opened up various avenues of research ^1^.Experimental design and biological interpretation of microbiome data has become an area of intense focus as the contributions to human health and disease are becoming clearer ^2, 3^. The ‘meta-omics’ approaches, such as metagenomics, metatranscriptomics and metaproteomics have been developed to study microbiomes without culturing and target the major macromolecules that constitute the community, namely DNA, RNA and proteins. While metagenomics (16S rRNA or whole genome sequencing) focuses on the taxonomy profile and functional potential ^4^, metatranscriptomics, metaproteomics and meta-metabolomics ^5^ uncover the functional response of the microbiome to stimuli on the short and long time-scale, respectively ^6, 7^.

Metatranscriptomics has been used to analyze microbial gene expression profiles from a variety of complex sample types, e.g. human microbiome, aquatic or terrestrial environments, plant-microbe interactions ^8^. Despite these applications, challenges still exist in the analysis of the complex metatranscriptomics data. Metatranscriptomics data is usually generated using high-throughput sequencing of short RNA-Seq reads using Illumina sequencing technology (PMID: 30298254). Many software tools and workflows are available for metatranscriptomics analysis. These include tools for RNA-Seq Data Preprocessing: Quality Control (FastQC), Ribosomal RNA removal (SortMeRNA, barrnap), host RNA removal (BMTagger), De Novo Assembly (Trinity, MetaVelvet, Oases, IDBA-MT, TAG), Transcript Taxonomy (Kraken, GOTTCHA, MetaPhlAn2), Functional Annotation (HUMAnN2), Annotation of assembled contigs are subjected to gene finding programs such as FragGeneScan followed by functional assignment using DIAMOND searches against KEGG, NCBI RefSeq, UniProt. Differential Expression analysis is performed by tools such as EdgeR, DeSeq2 and limma. “Reads-Based” analysis is performed by tools such as MetaTrans, COMAN, FMAP, SAMSA2, ASaiM and Assembly Based analysis: SqueezeMeta, IMP, MOSCA. Some of these open source tools ^9^ have been incorporated within the Galaxy bioinformatics workbench ^10^ to make it more accessible to users on a single platform.

**Figure 1.**
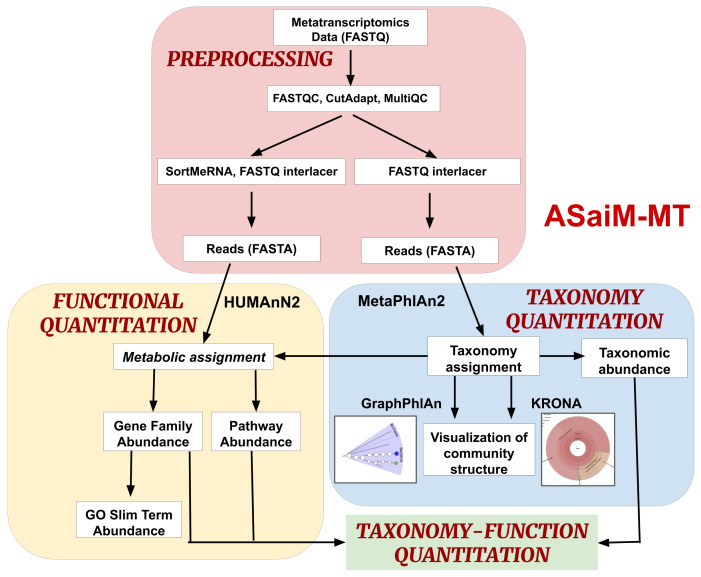
ASaiM-MT workflow: The workflow is divided into 4 parts. (i) Preprocessing: Process raw metatranscriptomics data to perform further analysis. (ii) Taxonomy Quantitation: Assignment of taxonomy along with abundance values and visualization. (iii) Functional Quantitation: metabolic assignment of identified functions and gene and pathway abundance annotation. (iv) Taxonomy-Function Quantitation: combine taxonomy and functional quantitation values into relative abundance values at different levels such as e.g., the abundance of a pathway between phyla.

ASaiM framework was previously developed by Batut *et al.* to perform metagenomics and metatranscriptomics data analysis ^11^. The major goal of ASaiM was to develop an accessible, resharable, and user-friendly framework for microbiome researchers, implemented within the Galaxy platform ^12^. The framework integrates a comprehensive set of microbiota related tools, predefined and tested workflows as well as supporting training material and documentation. It is available for users as a Docker image but also as a web server ( https://metagenomics.usegalaxy.eu/). This implementation also enables flexibility, so that the workflow can be customized for datasets of diverse origin as new software tools or methods emerge. To address the need for optimizing ASaiM for metatranscriptomics data, we added the ASaiM-metatranscriptomics (ASaiM-MT) ( Figure 1), a metatranscriptomics workflow, and rigorously tested it to ensure reliable analysis of metatranscriptomics data. Our testing and validation focused on using contemporary tools in their most current version ( Table 1), capable of handling large datasets, and ensuring that the outputs from each of the tools were compatible in order to build an integrated and automated workflow. The workflow also has potential for integration with other meta-omic tools and workflows in Galaxy, such as those designed for metaproteomics ^13^, to enable multi-omic data analysis.

**Table 1.** Enhancements in the ASaiM-MT workflow as compared to the original ASaiM shotgun metagenomics workflow.

Tool Function	ASaiM Shotgun Metagenomics	ASaiM-MT	Updates
**Quality control**	*FASTQC*	*FASTQC*	*Version change (0.69 → 0.72)*
		***MultiQC***	*Tool added*
**Adapter Trimming**	*TrimGalore!*	***CutAdapt***	*Tool replaced*
**Dereplication**	*VSearch*	*-*	
**rRNA selection**	*FilterwithSortmeRNA*	*FilterwithSortmeRNA*	*Version change (2.1b.4 → 2.1b.6)*
**Interlacing**	*FASTQ-Join*	***FASTQ interlacer***	*Tool replaced*
**Taxonomic assignation**	*MetaPhlAn2*	*MetaPhlAn2*	*No change*
**Formatting for the** **different taxonomic** **levels**	*Format MetaPhlAn2*	*Format MetaPhlAn2*	*No change*
**Functional assignation**	*HUMAnN2*	*HUMAnN2*	*No change*
**Visualization**	*Export to GraPhlAn*	*Export to GraPhlAn*	*Parameters changed*
	*Krona pie chart*	*Krona pie chart*	*Version change (2.6.1 → 2.6.1.1)*
	*GraPhlAn*	*GraPhlAn*	*No change*
	*Generation, personalization and * *annotation of tree*	*Generation, personalization and * *annotation of tree*	*No change*
**Regroup to GO terms**	*Group abundances*	***Group abundances***	*Tool updated*
**Unpack Pathway** **abundance**	*-*	***Unpack Pathway abundance to*** ***show gene families***	*Tool added*
**Extracting Gene level** **information**	*-*	***Create gene level families file***	*Tool added*
**Text manipulation tools**	*-*	***Select, Sort, Group***	*Tools added*

The ASaiM-MT workflow is available via the ASaiM framework, specially at https://metagenomics.usegalaxy.eu/), for users to test their metatranscriptomics data. It is supported by a step-by-step tutorial (link), available on the Galaxy Training Network (GTN) (citation), which provides explanation for the different steps and the opportunity for online, hands-on training in using the workflow, with a trimmed dataset.

## Methods

### Input

Our optimized ASaiM-MT workflow (available via https://metagenomics.usegalaxy.eu/) accepts Illumina paired end FASTQ sequence files (Forward read and Reverse read). As an alternative, a single-end FASTQ sequence can also be used as an input, with minor modifications in the downstream processing tool (such as changing the sequence type in CutAdapt and Filter with SortmeRNA as single end reads and also removing the FASTQ interlacer tool).

### Workflow implementation

The ASaiM-MT workflow contains all the processing steps and the parameters required for the metatranscriptomics analysis from RNA-Seq data collected under a single biological condition. This workflow is also compatible with the single-end sequencing reads although parameters have to be changed to accommodate this input. This workflow is a multi-step analysis with preprocessing/ data cleaning, taxonomy analysis and functional analysis. The starting data input for the workflow are the FASTQ files - forward and reverse reads (obtained from the Illumina sequencer). We describe below the tools and their functions in the workflow. For comparative analysis of multiple biological conditions, the users have an option to use the MT2MQ tool to generate inputs for statistical analysis (see discussion).

1) Preprocessing

**Figure 2.**
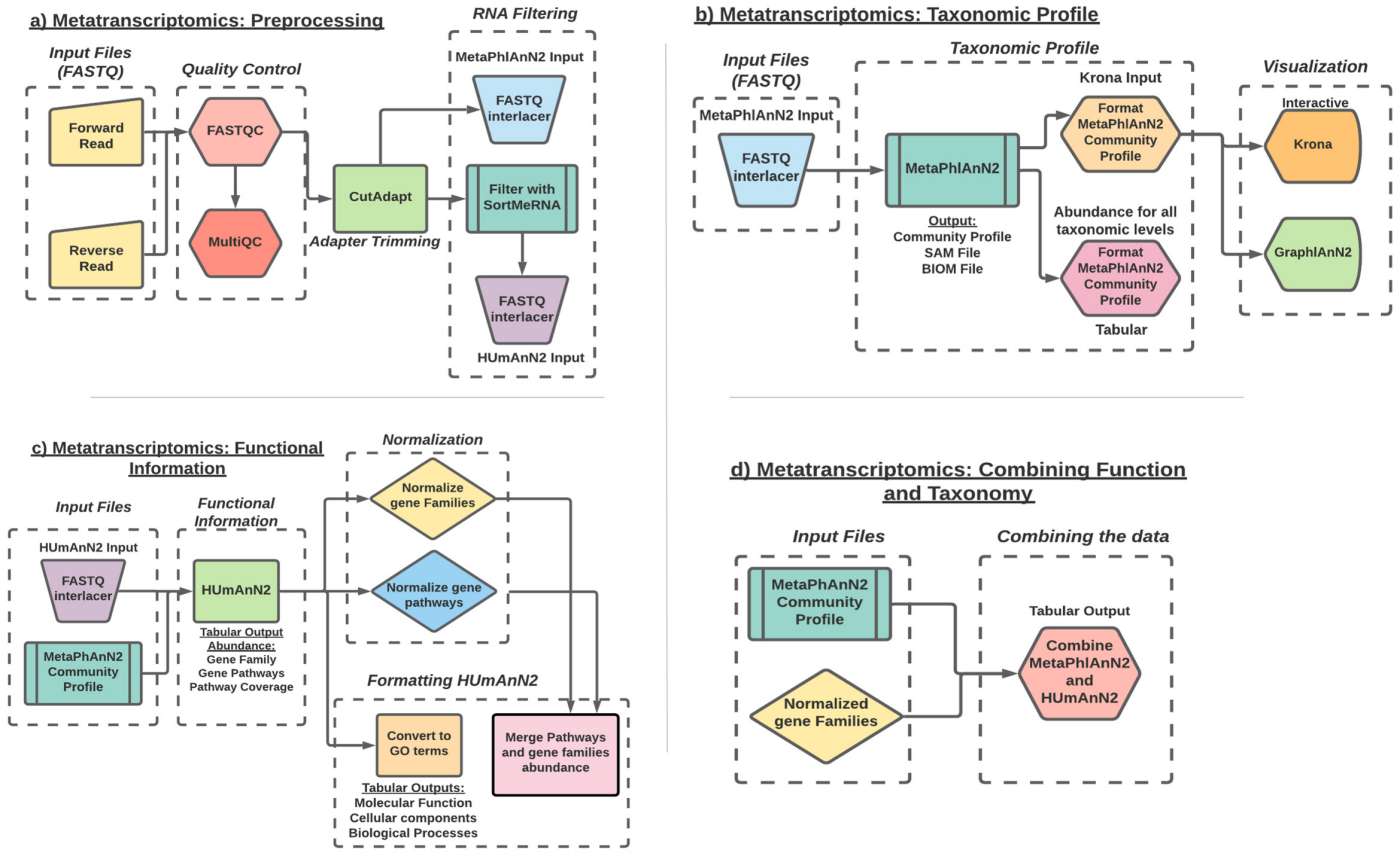
Diagram of the ASaiM-MT workflow: **a**) Preprocessing workflow: Workflow representation of the tools involved in quality check, data trimming and RNA filtering. **b**) Taxonomic profile workflow: workflow representation of taxonomy assignment tool (MetaPhlAn2) and post processing of the data using the Format MetaPhlAn2 tool. The workflow includes visualization of the data using interactive Krona and GraPhlAn plots. **c**) Functional information workflow: representation of tools involved in functional annotation (HUMAnN2), normalization of the data **d**) Combine Functional-Taxonomy abundance workflow: workflow representing tools that combines (Combine MetaPhlAn2 and HUMAnN2 outputs) and groups (Group abundances into GO slim terms) the functional and taxonomy output.

Occasionally, sequencing can introduce incorrect identification of nucleotides and these errors can lead to misinterpretation of the data, thus bringing in the need to preprocess ( Figure 2(a)) the data before analysis. The first step in our analysis is to perform quality control to remove such sequencing errors. For this, we use FastQC to assess the quality of each sample and MultiQC to combine each result into a single report. To improve the quality of the data, CutAdapt was used to trim low-certainty bases from reads, filter out reads of poor quality or short length, unwanted sequences, including adapters, primers, and poly-A tails. The ASaiM-metagenomics shotgun workflow uses Trim Galore! for trimming of adapters. Trim Galore! works as a wrapper that includes CutAdapt and FastQC. However, for the ASaiM-MT workflow we chose CutAdapt ^14^ for adapter trimming because it is more error tolerant, processes fast and modifies and filters reads according to user’s preference compared to TrimGalore!. Next, SortMeRNA ^15^ was used to remove any rRNA sequences, which are often used for easy taxonomic characterization of microbiomes but do not provide functional information. We eliminated the step of de-replication of reads (V-Search) in the ASaiM-MT workflow, in order to retain the multiple copies of sequences for metatranscriptomics quantitation. The final step in cleaning and processing the data is to interlace the forward and reverse reads since the following steps require a single file per sample. For performing this action, the original ASaiM Shotgun workflow used the FASTQ-joiner to join the reads. However, in the ASaiM-MT version, we use the FASTQ interlacer. FASTQ interlacer joins the forward (/1) and the reverse reads (/2) using the sequence identifiers; sequences without designation will be named as single reads. The reason ASaiM-MT uses FASTQ-interlacer rather than FASTQ-joiner is because the joiner tool combines the forward and reverse read sequence together while the interlacer puts the forward and reverse read sequences in the same file while retaining the entity of each read along with an additional file with unpaired sequences. We perform the interlacing on the data both before and after the SortMeRNA step since the following steps require both data with and data without rRNA.

2) Extraction of taxonomic profile

To understand a microbial community, we must first understand which organisms are present along with their abundance. There are several approaches to microbial taxonomic profiling, but this workflow ( Figure 2(b)) uses the marker gene approach. MetaPhlAn2 checks every read against a database of approximately one million clade-specific marker genes from nearly 17,000 reference genomes (bacterial, archaeal, viral, and eukaryotic) ^16^. For this particular step, we use all reads, including rRNA since they are useful for taxonomic profiling. The outputs of MetaPhlAn2 are a SAM (sequence alignment map) file and a BIOM (Biological Observation Matrix) file, which both show the mapping of reads onto the reference database, and Community Profile tabular output which contains information regarding the taxa present and their relative abundance. This table includes information at all taxonomic levels, so to parse it out into each separate level we use the Format MetaPhlAn2 tool.

We use two different tools to visualize the taxonomic profiles. First, we use Krona ^17^, which creates an interactive pie chart from the hierarchical taxonomic data. This chart is multi-layered for each taxonomic level and can be zoomed for viewing at each level. GraPhlAn ^18^ is the other visualization tool, which creates a publication-ready circular representation of a phylogenetic tree based on the taxonomic results. We must first use export2graphlan to convert the MetaPhlAn2 results to a format that GraPhlAn can use.

3) Extraction of functional information

After characterizing the taxonomic profile of each sample, we must determine which genes are expressed and the biological processes involved. To perform functional profiling ( Figure 2(c)), we use HUMAnN2 ^4^, which is a pipeline to quickly and accurately determine the presence and abundance of functional gene families and pathways from metagenomic or metatranscriptomics data.

For identifying the functions expressed by the community, we filter out the rRNA sequences, due to their high noise levels and the compute time needed for their analysis. The output of SortMeRNA and the identified community profile from MetaPhlAn2 helps HUMAnN2 to focus on the known sequences for the identified organisms. Software tools such as ‘Renormalize’, ‘Unpack Pathway abundance to show gene families’, ‘Create gene level families file’, ‘Group abundances’ and text manipulation tools such as ‘Select lines’, ‘group columns’ and ‘sort columns’ have been introduced in the ASaiM-MT workflow. This generates normalized abundances of gene families, gene pathways and GO slim terms present in each sample.

4) Combine taxonomic and functional information

The final step ( Figure 2(d)) in this analysis is to determine which microorganisms are contributing to the profile of functions indicated by the expressed RNA sequences. HUMAnN2 partially answers this question by including taxa in its gene family and pathway outputs, but it does not include the taxa's abundance, only the functional abundance. We can fill in this missing information with the MetaPhlAn2 results using the Combine MetaPhlAn2 and HUMAnN2 Outputs tools. This produces a table of functional terms and their abundances with the corresponding genus and species abundances for the taxa which contribute to said function via their expressed RNA sequences. The abundances are reported in RPK values (reads per kilobase), calculated as the sum of the scores for all alignments for a gene family. These alignment scores are calculated according to the number of matches of a specific sequence to its reference genome and further normalized to account for multiple reference genome matches.

## Results

**Figure 3.**
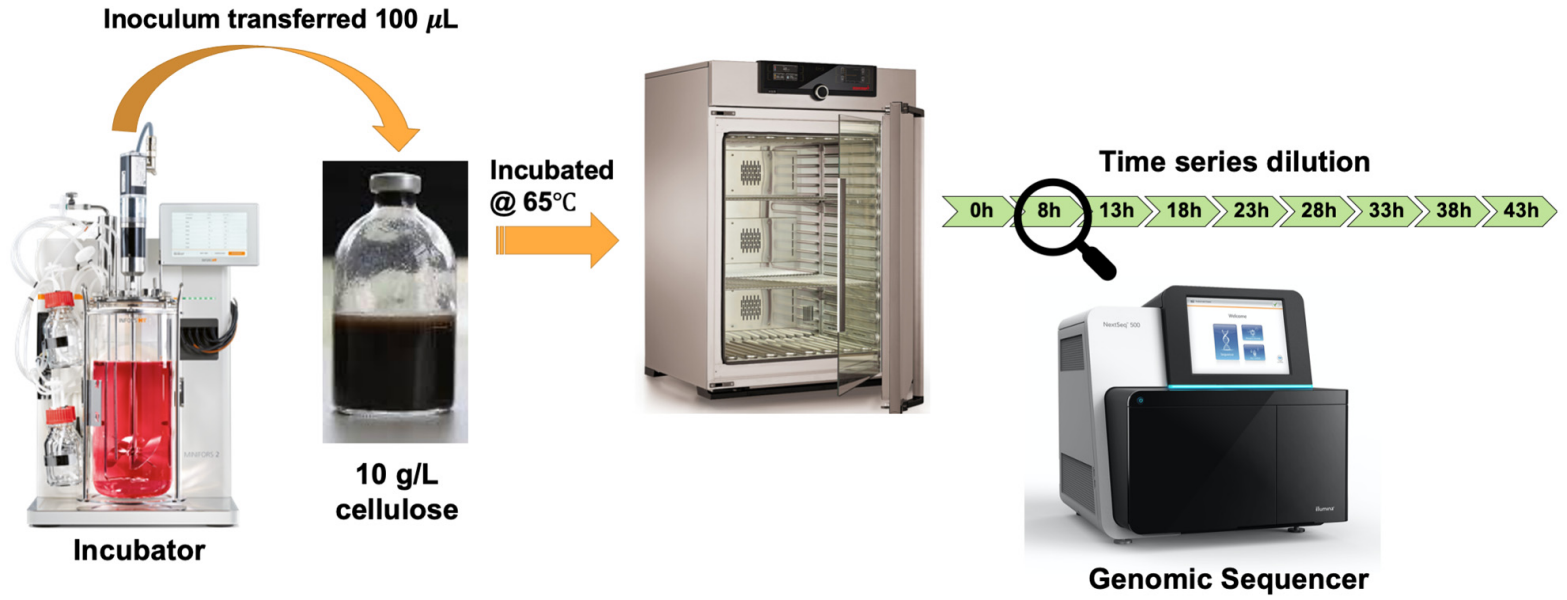
Graphical representation of Biogas reactor Dataset. A 100 µl inoculum was collected from a lab-scale biogas reactor incubated at 55 °C and transferred to an anaerobic flask containing 10 g/L of cellulose. Triplicate mRNA samples were taken in a time series from 0 to 43 hours after inoculation. Metagenomic and metaproteomic sequencing was performed for all time points. For this tutorial we used one of the triplicates (T1A) in the 8 hours’ time point.

To demonstrate the use of the ASaiM-MT workflow, we analyzed a representative metatranscriptomic data set obtained from a microbial community within a thermophilic biogas reactor ^19^ which digests municipal food waste and manure ( Figure 3). The microbial community was sampled from the bioreactor and transferred to a rich medium containing lignocellulose from Norwegian Spruce and incubated at 65°C as an enrichment strategy. Triplicate mRNA samples were taken in a time series from 0 to 43 hours after inoculation. The mRNA was sequenced for paired-end reads (2 x 125 bp) on one lane of an Illumina HiSeq 3000. For the purpose of this study, we took only one time point (8hr). The paired FASTQ files (forward and reverse reads) were then subjected to the ASaiM-MT workflow ( Figure 2).

The ASaiM-MT workflow consists of four steps, i) preprocessing of the data, ii) extraction of community profile, iii) extraction of functional information, and iv) combining taxonomic and functional information. The data is preprocessed to make it compatible for MetaPhlAn2 (taxonomy) and HUMAnN2 (Function) annotation of the data.

**Figure 4.**
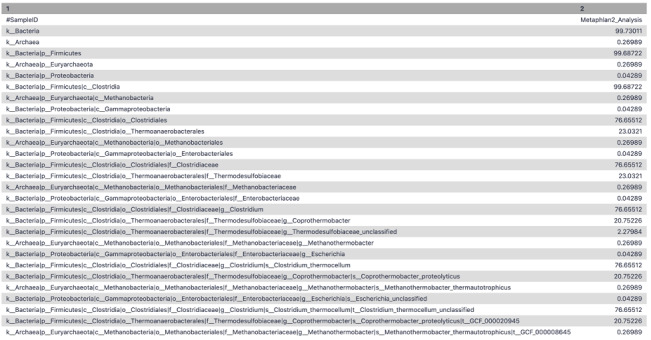
MetaPhlAn2 community profile. A tabular representation of MetaPhlAn2 community profile displaying the different levels of taxonomic classification and its relative abundance at that level.

To extract the taxonomic profile, the MetaPhlAn2 suite was run on the adapter trimmed interlaced files. The Community Profile output contained the information regarding the microbiome community present in the sample along with its relative abundance at different levels, i.e., Kingdom, Phylum, Class, Order, Family, Genus, Species, and Strain ( Figure 4). For example - (k__Bacteria|p__Firmicutes|c__Clostridia|o__Thermoanaerobacterales|f__Thermodesulfobiaceae|g__Coprothermobacter|s__Coprothermobacter_proteolyticus) states that *kingdom* is bacteria, *class* is clostridia, belonging to *order* thermoanaerobacter, *family* of thermodesulfobiaceae, *genus* is copthermobacter and *species* is *Comprothermobacter proteolyticus*. As it is a cellulose-degrading consortium from anaerobic digestion, *Coprothermobacter* and *Clostridium* were expected to be identified for this dataset, demonstrating the accuracy of these tools.

**Figure 5.**
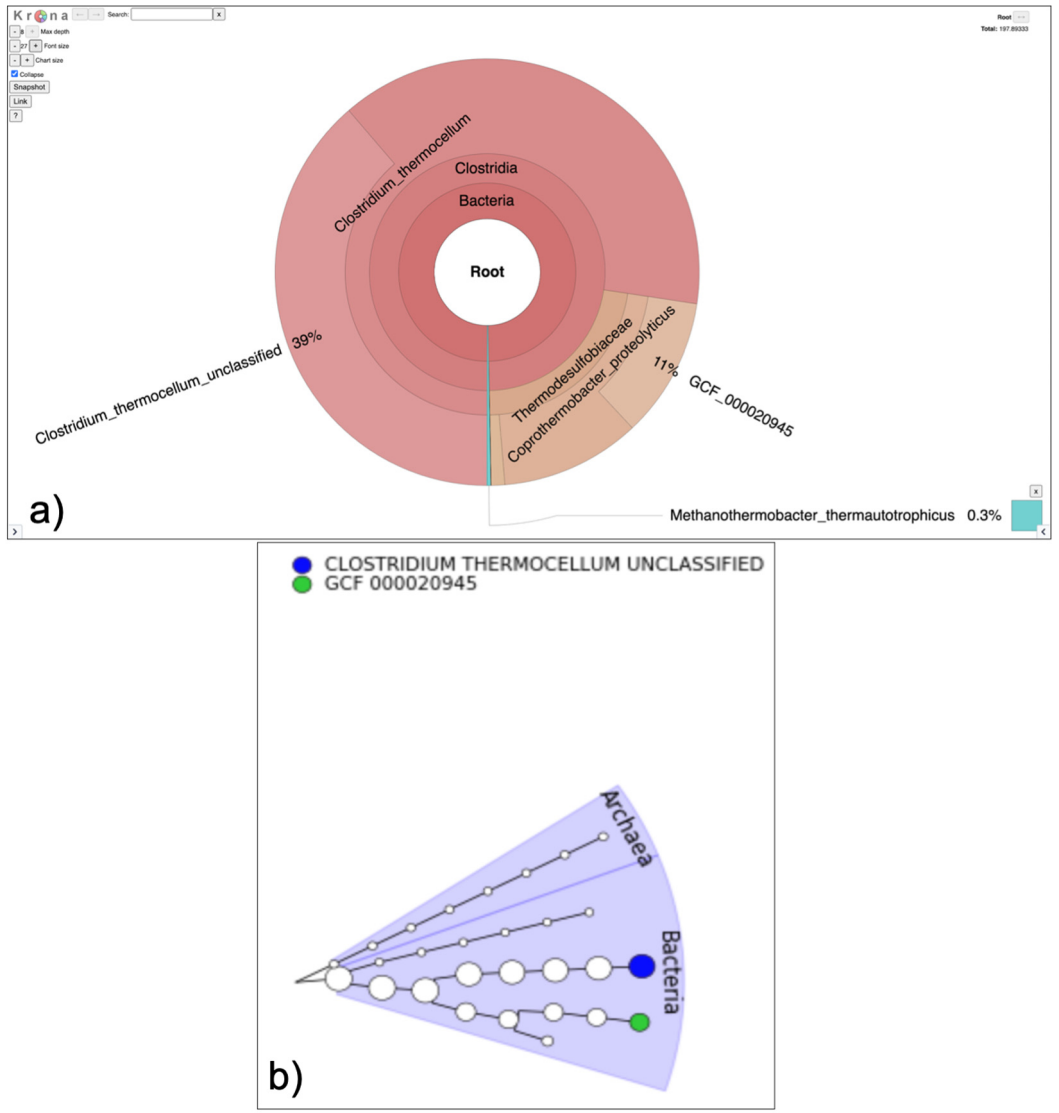
Visualization of Taxonomy output. **a**) The Krona output provides interactive representation of the community in the sample. In this case *Coprothermobacter* and *Clostridium* were the most abundant genera. **b**) The GraPhlAn outputs circular phylogenetic trees showing that Archaea and bacteria are present in the sample.

The community profile is further processed using the Format MetaPhlAn2 tool which splits the MetaPhlAn2 output and categorizes them into various taxonomy levels (Kingdom, Phylum, Class, Order, Family, Genus, Species) with corresponding abundance values. Supplementary Figure S1 ( *Extended data* ^20^) shows genus level relative abundance values associated with genera present in the dataset. For visualization of the taxonomic profile, GraPhlAn and Krona (interactive) were used ( Figure 5a and 5b).

The HUMAnN2 suite of tools were used to extract functional information along with their relative abundance (RPK). HUMAnN2 provides 3 different outputs- Gene family and their abundance ( *Extended data*: Supplementary Figure S2a ^20^), pathways and their abundance ( *Extended data*: Supplementary Figure S2b ^20^) and pathway and their coverage ( *Extended data*: Supplementary Figure S2c ^20^).

In this workflow, the UniRef50 database was used to classify the gene family, but the users also have a choice to use UniRef90. Gene family abundance at the community level is stratified to show the contributions from known and unknown species. The gene family output shows total abundance value which is the sum total of individual species abundance values (reported as RPK values). Additionally, the tabular output also enlists the contribution of individual species to the gene family abundance (Extended data: Supplementary Figure S3 ^20^). While there are some applications, e.g., strain profiling, where RPK units are superior to depth-normalized units, most of the time we need to renormalize our samples prior to downstream analysis. The gene families can be a long list of IDs and going through the gene families one by one to identify the interesting ones can be cumbersome and error prone. To help construct “the bigger picture”, we could identify and use categories of genes using the gene families. Gene Ontology (GO) analysis is widely used to reduce complexity and highlight biological processes in genome-wide expression studies. There is a dedicated tool called Group abundances of UniRef50 gene families obtained to gene ontology (GO) slim Terms, which groups and converts UniRef50 gene family abundances generated with HUMAnN2 into GO slim terms (Extended data: Supplementary Figure S4 ^20^) as the name suggests.

The functional and taxonomic annotations from MetaPhlAn2 and HUMAnN2 are further normalized and combined to create a single tabular output.

## Use cases

Here we provide a trimmed version of the biogas reactor dataset to demonstrate the use of the ASaiM-MT workflow in the tutorial available in the GTN.

*Link*: https://training.galaxyproject.org/training-material/topics/metagenomics/tutorials/metatranscriptomics/tutorial.html

*Trimmed input*: https://zenodo.org/record/3362849

*Workflow*: https://training.galaxyproject.org/training-material/topics/metagenomics/tutorials/metatranscriptomics/workflows/

## Discussion

The ASaiM-MT workflow is made available in the Galaxy platform via ASaiM, enabling accessibility and flexibility for customization. There are a few tools that can be used alternatively or in addition to the existing tools. The ASaiM-MT workflow (as mentioned in the methods section) was tested to ensure that the workflow works on metatranscriptomics datasets. For details about default parameters used, we recommend visiting the metatranscriptomic tutorial available on the GTN material ^21^.

For example, in ASaiM-MT, we map the UniRef 50 values to GO terms, but they can be also mapped to the MetaCyc reactions ^22^, KEGG Orthogroups (KOs) ^23^, Pfam domains ^24^, EC categories ^25^ and EggNOG (including COGs) ^26^ using the HUMAnN2 regroup tool. A current limitation of the ASaiM-MT workflow is that it can process only one paired-end or single-end data at a time. In order to generate an input for statistical analysis, we have developed an additional post-processing workflow and Galaxy tool called MT2MQ ( https://github.com/galaxyproteomics/tools-galaxyp/tree/master/tools/mt2mq), which integrates results from multiple outputs from the ASaiM-MT workflow. The MT2MQ workflow combines the gene abundance output from multiple samples or conditions, normalizes the values and makes it compatible with statistical tools such as metaQuantome tool ^13^, which can be used for visualizing and interpreting results. Furthermore, we are in the process of developing tools that can help perform multi-omics studies by integrating results from the ASaiM-MT workflow to our existing metaproteomics workflows.

## Conclusion

ASaiM-MT workflow is a robust and extensible Galaxy workflow which is now optimized and tested for metatranscriptomics data. The workflow consists of tested open-source tools in the area of RNA sequence analysis, such as SortMeRNA, MetaPhlAn2 and HUMAnN2. ASaiM-MT in Galaxy offers users a high-level control over their data and provides different analysis options. The GTN offers documentation and resources necessary for new users to gain mastery in using this workflow and associated tools for data analysis for their research projects. ASaiM-MT allows users to not only understand the taxonomy but also the functional composition and pathways expressed by the microbiome present in diverse microbial communities of interest. 

## Data availability

### Underlying data

Zenodo: Training Data for “Metatranscriptomics analysis using microbiome RNASeq data”, http://doi.org/10.5281/zenodo.3362849 ^27^.

### Extended data

Zenodo: Supplementary for ASaiM-MT: A validated and optimized ASaiM workflow for metatranscriptomics analysis within Galaxy framework, http://doi.org/10.5281/zenodo.4341391 ^20^.

This project contains the following extended data:

- Supplementary Figure S1: MetaPhlAn2 Genus-Level Abundance
- Supplementary Figure S2a: HUMAnN2 Gene Family Abundance
- Supplementary Figure S2b: HUMAnN2 Pathway Abundance
- Supplementary Figure S2c: HUMAnN2 Pathway Coverage
- Supplementary Figure S3: Uniref50 Gene Family output with abundance
- Supplementary Figure S4: Conversion of Uniref 50 values to GO terms

Data are available under the terms of the Creative Commons Attribution 4.0 International license (CC-BY 4.0).

## Software availability 

Software available from: https://metagenomics.usegalaxy.eu/

Source code available from: https://github.com/ASaiM/framework

Archived source code at time of publication: http://doi.org/10.5281/zenodo.4455627 ^28^.

License: Apache 2 License

Docker: https://quay.io/repository/bebatut/asaim-framework (command: docker pull quay.io/bebatut/asaim-framework).
