# Supplementary material for: ASaiM-MT: a validated and optimized ASaiM workflow for metatranscriptomics analysis within Galaxy framework
Source: F1000Res. 2021 Apr 19;10:103. Originally published 2021 Feb 11. [Version 2] doi: 10.12688/f1000research.28608.2 (PMC8383124; doi:10.12688/f1000research.28608.2)
Supplement: Peer review history

# Peer review: Reviewer response for version 2

**DOI:** 10.5256/f1000research.55709.r90589

**Published:** 2021-08-23

**Authors:** Won Kyong Cho

**Affiliations:** 1 Research Institute of Agriculture and Life Sciences, College of Agriculture and Life Sciences, Seoul National University, Seoul, South Korea

In this study, the authors provided a new workflow referred to as ASaiM for metatranscriptomics. The introduction was easy to understand the background of the study. Results and methods were written in detail. Figures and tables are very good to understand the workflow of the ASaiM-MT. In addition, the authors provided a data set for the demonstration. Overall, the quality of the manuscript is high enough for publication as it is. I hope the newly developed platform can be usefully used by many biologists working with microbiomes.

Is the rationale for developing the new method (or application) clearly explained?

Yes

Is the description of the method technically sound?

Yes

Are the conclusions about the method and its performance adequately supported by the findings presented in the article?

Yes

If any results are presented, are all the source data underlying the results available to ensure full reproducibility?

Yes

Are sufficient details provided to allow replication of the method development and its use by others?

Yes

Reviewer Expertise:

Metagenomics, metatranscriptomics, viromes

I confirm that I have read this submission and believe that I have an appropriate level of expertise to confirm that it is of an acceptable scientific standard.

# Peer review: Reviewer response for version 2

**DOI:** 10.5256/f1000research.55709.r83478

**Published:** 2021-04-27

**Authors:** Caitlin Simopoulos

**Affiliations:** 1 Department of Biochemistry, Microbiology and Immunology, University of Ottawa, Ottawa, ON, Canada

The authors have revised the manuscript to address my concerns, and I am satisfied with the revisions. I believe the re-formatting of the Methods section helps with comprehension.

 I enjoyed the figures describing the method and feel like they add to the manuscript. Inclusion of the Galaxy workflow will also help users analyze their own data using this tool. I also appreciated the links to a separate tutorial and the Gitter community. This approach will help guide and welcome new users to the tool.

 Some minor suggestions are to correct two typos:

1. In the intro: "Functional Annotation ( *HUMAnN2* ^7^), Annotation of assembled contigs are subjected to gene finding programs such as *FragGeneScan* ^2" ^where I believe the "," should be a ".".
2. In Figure 3 legend, I believe "metaproteomics" or "metagenomics" should be "metatranscriptomics". This may be more important to correct.

Is the rationale for developing the new method (or application) clearly explained?

Partly

Is the description of the method technically sound?

Partly

Are the conclusions about the method and its performance adequately supported by the findings presented in the article?

Partly

If any results are presented, are all the source data underlying the results available to ensure full reproducibility?

Yes

Are sufficient details provided to allow replication of the method development and its use by others?

Yes

Reviewer Expertise:

Computational biology, bioinformatics, RNA-sequencing data pipeline development and analysis, microbiomes

I confirm that I have read this submission and believe that I have an appropriate level of expertise to confirm that it is of an acceptable scientific standard.

# Peer review: Reviewer response for version 1

**DOI:** 10.5256/f1000research.31653.r79463

**Published:** 2021-02-18

**Authors:** Caitlin Simopoulos

**Affiliations:** 1 Department of Biochemistry, Microbiology and Immunology, University of Ottawa, Ottawa, ON, Canada

The authors describe an optimized workflow for metatranscriptomic microbial community data named **ASaiM-MT**. **ASaiM-MT** is an extension of **ASaiM**, a workflow mostly focused on metagenomic data available via Galaxy. Using helpful diagrams, the authors describe how **ASaiM-MT** differs from the original **ASaiM** workflow, and detail which tools from the **Galaxy** platform are used. Finally, the authors use data from a biogas reactor study the pipeline and demonstrate results that can be expected using **ASaiM-MT**. I particularly enjoyed the fact that the article is accompanied by written training material for the workflow. I believe that tutorials and user-friendly tools expand the audience of traditionally “bioinformatician-only” tools.  However, I have a few suggestions for the authors to strengthen their presentation of the metatranscriptomic workflow before publication.   

**Major comments:**

1. In your discussion, you highlighted a major limitation of **ASaiM-MT**: the fact that it can only handle a single sample and does not complete comparative analysis. However, you also mention that you have developed post-processing tools for this reason. It might be good to highlight that **ASaiM-MT** is essential (in particular for data pre-processing and identification of functional and taxonomic information) to be completed before statistical analysis. In addition, is it possible to process data in ASaiM-MT in batches? This might also lessen the perceived limitation of the tool.
2. Sometimes it is not completely clear why **ASaiM-MT** is needed if **ASaiM** was developed for both meta-genomics and -transcriptomics. It is important to be specific and highlight the rationale for developing the **ASaiM-MT **workflow. An example *:* *“For performing this action, the original ASaiM Shotgun workflow used the FASTQ-joiner to join the reads. However, in the ASaiM-MT version, we use the FASTQ interlacer. FASTQ interlacer joins the forward (/1) and the reverse reads (/2) using the sequence identifiers; sequences without designation will be named as single reads. The reason ASaiM-MT uses FASTQ-interlacer rather than FASTQ-joiner is because the joiner tool combines the forward and reverse read sequence together while the interlacer puts the forward and reverse read sequences in the same file while retaining the entity of each read along with an additional file with unpaired sequences.” * Was this change made because **FASTQ interlacer** is more appropriate for RNA sequencing, or was it a change to improve on the original **ASaiM** workflow?
3. The abstract and introduction seem to emphasize the importance of studying the human microbiome and its connections to health and well-being, however, this manuscript uses a microbial community obtained from a biogas reactor. This is a very interesting community that warrants further research, but the introduction does not match the sample of interest. I suggest updating the introduction to focus less on the human microbiome and emphasize the importance of studying microbial communities in general. Alternatively, if you’d like to focus on human microbiomes, the example data used should reflect the human focus.
4. In your discussion, you say: *“* *There are a few tools that can be used alternatively or in addition to the existing tools.”* Does this mean that there are other tools like **ASaiM-MT** that can be used? Your introduction mainly listed tools that complete specific tasks and not an end to end workflow. If there are other workflow tools, a comparison should be highlighted in the discussion or introduction. What makes **ASaiM-MT** unique compared to other tools/workflows? What are its strengths? Is it more user-friendly than other tools? Expanding on this will help strengthen your conclusions about the tool itself.

**Minor comments:**

1. There are sections that can read like a “grocery list” of software names (eg. second paragraph of the introduction). I understand why you are listing them, but it could be useful to emphasize that each of these tools performs a single task and need to be put together into a workflow for a complete experiment. These tools are also often missing citations.
2. It can be difficult to understand which words are actually software names. Is it possible to type them in a monospace font? Or to bold the names?
3. At times the “Methods” section can get confusing, particularly when MT is being compared to the original **ASaiM.** My suggestion is to include numbers or roman numerals in the “in between” steps as described in Figure 2. That way you can also reference them in the text. For example, in “a) Preprocessing” there would be i. Input files, ii. Quality Control, iii. Adapter Trimming… etc..  It might also be useful to include these headers (and numbers/letters) in your Methods section to let readers follow along.
4. “PMID 30298254” is used instead of a citation

Is the rationale for developing the new method (or application) clearly explained?

Partly

Is the description of the method technically sound?

Partly

Are the conclusions about the method and its performance adequately supported by the findings presented in the article?

Partly

If any results are presented, are all the source data underlying the results available to ensure full reproducibility?

Yes

Are sufficient details provided to allow replication of the method development and its use by others?

Yes

Reviewer Expertise:

Computational biology, bioinformatics, RNA-sequencing data pipeline development and analysis, microbiomes

I confirm that I have read this submission and believe that I have an appropriate level of expertise to confirm that it is of an acceptable scientific standard, however I have significant reservations, as outlined above.

# Response

**Published:** 2021-03-25

**Authors:** Subina Mehta

**Affiliations:** University of Minnesota, USA

We greatly appreciate the reviewer’s comments and suggestions. We have incorporated the required changes in the updated version of the manuscript.

**Major comments:**

**1. In your discussion, you highlighted a major limitation of ASaiM-MT: the fact that it can only handle a single sample and does not complete comparative analysis. However, you also mention that you have developed post-processing tools for this reason. It might be good to highlight that ASaiM-MT is essential (in particular for data pre-processing and identification of functional and taxonomic information) to be completed before statistical analysis. In addition, is it possible to process data in ASaiM-MT in batches? This might also lessen the perceived limitation of the tool.**

**A:** *We would like to thank the reviewer for the comment. We have edited the abstract and mentioned that ASaiM-MT is essential for preprocessing and  identification of taxonomy and functional information before performing comparative analysis. Also, Galaxy has a function of providing datasets as a collection rather than a single input which can be used to handle multiple datasets. In the Galaxy platform, we have other software tools such as MT2MQ and metaQuantome, which can perform comparative statistical analysis.*

**2. Sometimes it is not completely clear why ASaiM-MT is needed if ASaiM was developed for both meta-genomics and -transcriptomics. It is important to be specific and highlight the rationale for developing the ASaiM-MT workflow. An example: “For performing this action, the original ASaiM Shotgun workflow used the FASTQ-joiner to join the reads. However, in the ASaiM-MT version, we use the FASTQ interlacer. FASTQ interlacer joins the forward (/1) and the reverse reads (/2) using the sequence identifiers; sequences without designation will be named as single reads. The reason ASaiM-MT uses FASTQ-interlacer rather than FASTQ-joiner is because the joiner tool combines the forward and reverse read sequence together while the interlacer puts the forward and reverse read sequences in the same file while retaining the entity of each read along with an additional file with unpaired sequences.”  Was this change made because FASTQ interlacer is more appropriate for RNA sequencing, or was it a change to improve on the original ASaiM workflow?**

**A:** *Thank you for this comment. We replaced the FASTQ-joiner tool with the FASTQ interlacer to improve on the original workflow. The FASTQ interlacer tool maintains the integrity of reads by maintaining the forward and the reverse sequence identifiers, as compared to joining them into a single read file, as is done by  FASTQ-joiner tool.*

**3. The abstract and introduction seem to emphasize the importance of studying the human microbiome and its connections to health and well-being, however, this manuscript uses a microbial community obtained from a biogas reactor. This is a very interesting community that warrants further research, but the introduction does not match the sample of interest. I suggest updating the introduction to focus less on the human microbiome and emphasize the importance of studying microbial communities in general. Alternatively, if you’d like to focus on human microbiomes, the example data used should reflect the human focus.**

**A:** *We thank the reviewer for the comment and have edited the abstract and introduction to highlight the importance of microbiome research in ecology as well as clinical research.*

****

**4. In your discussion, you say: “There are a few tools that can be used alternatively or in addition to the existing tools.” Does this mean that there are other tools like ASaiM-MT that can be used? Your introduction mainly listed tools that complete specific tasks and not an end to end workflow. If there are other workflow tools, a comparison should be highlighted in the discussion or introduction. What makes ASaiM-MT unique compared to other tools/workflows? What are its strengths? Is it more user-friendly than other tools? Expanding on this will help strengthen your conclusions about the tool itself.**

**A:** *We thank the reviewer for the comment. The tools mentioned in the discussion offer an alternative option to the tools that we have used in the AsaiM-MT workflow,  especially if the users have a preference. However, although we consider these tools to be appropriate to metatranscriptomics research, we have not tested these tools.*

*ASaiM-MT workflow, due to its availability in Galaxy, offers an user-friendly option to the existing command line tools. The ASaiM-MT workflow has been tested with different datasets to ensure its compatibility. There is a systematic documentation available for the usage of the workflow in the Galaxy Training Network (GTN). Users can also ask questions to developers and users via the Gitter channel, if needed.*

****

**Minor comments:**

**1. There are sections that can read like a “grocery list” of software names (eg. second paragraph of the introduction). I understand why you are listing them, but it could be useful to emphasize that each of these tools performs a single task and need to be put together into a workflow for a complete experiment. These tools are also often missing citations.**

**A:** *Thanks to the reviewer for the comment. The tools listed are alternatives to the existing tools in the workflow and have not been incorporated as a workflow. We have added citations to these tools.*

****

**2. It can be difficult to understand which words are actually software names. Is it possible to type them in a monospace font? Or to bold the names?**

**A:** *Thanks to the reviewer for this comment. I have formatted the tool names by making them italic.*

**3. At times the “Methods” section can get confusing, particularly when MT is being compared to the original ASaiM. My suggestion is to include numbers or roman numerals in the “in between” steps as described in Figure 2. That way you can also reference them in the text. For example, in “a) Preprocessing” there would be i. Input files, ii. Quality Control, iii. Adapter Trimming… etc..  It might also be useful to include these headers (and numbers/letters) in your Methods section to let readers follow along.**

**A:** *Thank you for the comment. I have reformatted the method section according to Figure 2.*

****

**4. “PMID 30298254” is used instead of a citation**

**A:** *Thank you reviewer for pointing this out. I have made the required change.*
